# Editorial: Exploring plant-based therapies in veterinary medicine

**DOI:** 10.3389/fvets.2025.1719496

**Published:** 2025-11-28

**Authors:** Narda Gail Robinson

**Affiliations:** CuraCore VET, Fort Collins, CO, United States

**Keywords:** veterinary, herbal, botanical medicine, traditional Chinese medicine, cannabis, phytomedicinal, veterinary medicine, herbal medicine

## The evolution of botanical medicines, with cannabis an emerging leader

Prior to the rise of the pharmaceutical industry in the early 20th century, plant-based remedies constituted mainstream care for both humans and animals. Even cannabis was listed in the United States Pharmacopeia (USP) for nearly a century (1850–1942), legitimizing, albeit temporarily, its medicinal value for a diverse assortment of medical maladies.

In the early 1900s, synthesized pharmaceuticals overshadowed phytomedicinals. Herbal medicine saw its status degraded to something akin to folklore. The lack of adequate regulatory control of patent medicines and “snake oils” created a “buyer beware” climate. Compared to industry-made drugs, herbal mixtures lacked quality control, regulatory oversight, batch-to-batch consistency, and more. Unknown mechanisms of action, risk-benefit ratios, proper dosing, interactions, etc. were far greater in number with “natural” products than a pill produced in a lab. Prescribing botanical medicine for animals large and small, compared to humans, introduces even greater challenges with dosing, metabolism, and toxicity in the target species. And yet, shelves in big-box pet stores and even veterinary hospitals contain a multitude of herbal supplements. Companies may claim that their products improve digestive health, support internal organ function, relieve pain, eliminate itching, and so on. When pressed, however, they may have no published evidence of safety or effectiveness and no independent testing to verify their contents and quality.

To help move the field forward in a rational, evidence-based manner, we are pleased to present this first issue for the Research Topic, “*Exploring Plant-Based Therapies in Veterinary Medicine*.” The papers it includes range from cell-based studies to clinical trials and incorporate species ranging from dogs and cats to dairy cows and Simmental bulls.

For example, Gaudio et al. studied the pharmacologic and toxicologic properties of thymol, a compound found in essential oils such as thyme, basil, oregano, and bergamot. Thymol exerts broad-spectrum antimicrobial activity against both Gram-positive and Gram-negative bacteria, which explains its inclusion in oral care and dermatologic formulae. Thymol confers antiviral activity against feline calicivirus, feline coronavirus, and norovirus. It even helps battle fungal and parasitic diseases. Physiologically, thymol counters oxidative stress, reduces inflammation, and modulates immune function. In livestock diets, it improves weight gain, feed efficiency, and immune function. In this systematic review, Pérez Gaudio et al. add to the knowledge base on thymol's relevance in veterinary health care.

Moving on to metabolomics, Li et al. studied the effects of a Traditional Chinese Medicine (TCM) formula, Guiqi Yimu Powder (GYP), on an *in vitro* fatty liver cell model of dairy cows. Fatty liver disease typically affects up to a third of high-yielding dairy cows in the periparturient period. It lowers resistance to disease and increases the risk of mastitis, hoof problems, and reproductive conditions while also compromising the quality and yield of their milk production. Through integrated transcriptomics, proteomics, metabolomics, and network pharmacology, Li et al. provided insight into how GYP impacts glutathione cycling in fatty liver cells, suggesting potential therapeutic applications for dairy cows with fatty liver disease.

Chen et al. studied a TCM formula in Simmental bulls with oligoasthenozoospermia (OA), characterized by reduced sperm count and motility. They adapted their “Modified Yougui Powder,” or MYP, from the classical remedy, Yougui Pill. They found that MYP administration in bulls with OA improved sperm count and motility, regulated hormone levels, and restored amino acid metabolic homeostasis. In so doing, they not only identified a cost-effective therapeutic option for OA; they also demystified the mechanisms of action of a TCM formula, paving the way to modernizing the practice of Chinese medicine.

Confronted by a diagnosis of cancer in their animal, veterinary clients frequently ask if herbs can cure cancer. While plants cannot replace chemotherapy, radiation therapy, or surgery, emerging evidence suggests that some herbs, such as mistletoe, may have anticancer effects, but far more research is warranted. Cakiroglu et al. compared two extracts of mistletoe on cells from an aggressive type of oral cancer in cats, feline oral squamous cell carcinoma (FOSCC). Based on a study of FOSCC cells, they showed that mistletoe extract disrupts key processes of cancer cell proliferation and survival.

In the realm of digestive disorders, about 20% of dogs develop chronic inflammatory enteropathy (CIE), manifesting as vomiting and diarrhea triggered by certain foods, bacteria, or immunologic hyperreactivity in the gut. Over time, ongoing inflammation erodes surface integrity in the intestinal lining, allowing endotoxins to enter the bloodstream. Móritz et al. explored the anti-inflammatory, antimicrobial, and antioxidant properties of flavonoids such as luteolin, quercetin, and grape seed extract oligomeric proanthocyanidins (GSOP). They confirmed that specific flavonoids could reduce reactive oxygen species levels and tumor necrosis factor alpha (TNF-alpha). These findings indicate that flavonoids may assist in the management of CIE in dogs, but further studies are needed prior to clinical implementation.

Bharani et al. also looked at canine gastrointestinal health issues, this time through protecting and modulating the gut microbiome. They studied the adaptogenic and anti-inflammatory effects of ashwagandha root extract (ARE) on the microbiome, finding that ARE improved certain hematologic and biochemical profiles in healthy Beagles. More studies are required to help us grapple with the vast unknowns about the gut microbiome, which some classify as an “invisible organ.”

Canine infectious respiratory disease (CIRD) and canine pneumonia outbreaks spiked in North America in late 2023, raising public awareness of its risks and transmissibility. CIRD has a variety of etiologies, including canine adenovirus type 2, canine influenza virus, and canine herpesvirus type 1, as well as secondary bacterial infections with S*treptococcus* and *Bordetella bronchiseptica*. Anxieties about antibiotic resistance and side effects of long-term steroid treatment have prompted the search for new therapeutic strategies that lessen morbidity and hasten healing. To this end, Ji et al. conducted a prospective, randomized, controlled trial on an extract (Anemoside B4 or AB4) of pulsatilla. Compared to placebo, dogs receiving an AB4 injection had faster recovery times, lower composite clinical scores, and a significantly higher cure rate with no serious adverse reactions or mortality observed.

Finally, the cannabinoid study by Wang et al. illustrates the strides that veterinary botanical medicine has made over the past decade since the passage of the 2014 Farm Bill in the United States. This bill, officially named the Agricultural Act of 2014, authorized academic and agricultural institutions to perform research on industrial hemp. This shift in federal policy laid the groundwork for studies on veterinary usage by creating a legal path forward.

In the paper by Wang et al., researchers compared the absorption of various cannabinoid preparations: (1) a CBD isolate, (2) a cannabidiolic acid (CBDA) isolate, (3) a CBDA full-spectrum formula, and (4) a combined CBD/CBDA full-spectrum formula. They found that CBDA exhibited better absorption than CBD, whether ingested in isolate or full-spectrum form.

Studies on cannabis are building a more complete picture of the mechanisms, pharmacokinetics, clinical applications, and adverse effects of cannabis derivatives. In the short amount of time since cannabis research has become legal, we now have far more information on hemp-derived cannabinoids in veterinary medicine than for any other botanical medicine product. Furthermore, the cannabis industry has modeled, through its generation of certificates of analysis (CoA), how to give consumers transparency about product purity, quality, safety, and contaminants. Why, we might ask, are manufacturers of all botanical remedies not following suit?

